# Translation of individualized medicine into clinics and the discourse of “hypothetical ethics”

**DOI:** 10.1186/1878-5085-5-S1-A104

**Published:** 2014-02-11

**Authors:** Martin Langanke, Tobias Fischer

**Affiliations:** 1 Ernst-Moritz-Arndt-Universität Greifswald, Theologische Fakultät,Greifswald, Am Rubenowplatz 2-3, 17489 Greifswald, Germany; 2 Universitätsmedizin Greifswald, Department für Ethik, Theorie und Geschichte der Lebenswissenschaften, c/o Institut für Geschichte der Medizin, Greifswald, Germany

## 

The analysis of the ethical discourse on Individualized Medicine (IM) within the context of PPPM, shows that the most extensively studied areas are (still) relatively far away from a practical implementation. The discussion of IM as an engine of a "dictatorship of prevention", in which IM is placed in the context of the ethical acceptability of mandatory screening tests or the rejection of the principle of solidarity under the banner of personal health responsibility, can be understood as a prime example. However, such discussions are situated within the range of a purely "hypothetical ethics", since the extensive medical knowledge and applications they are based on merely exist in the anticipation of potential successes, rather than the outcome of the real research context of IM, which - particularly in the area of complex diseases - does not go far beyond the level of rather trivial recommendations for a fundamentally healthy lifestyle. On the other hand, if one focuses on the reality in the field of IM research, it becomes evident that there are promising approaches mainly in two areas, both of which are not very promising for "hypothetical ethics": The fields of biomarker-based prediction of the course of a disease (e.g. in oncology) and the biomarker-based prediction of the success of drug and non-drug interventions. The progress in research in Germany's largest joint project for IM, *GANI_MED – Greifswald Approach to Individualized Medicine* – exactly points in that direction: The promising studies within GANI_MED refer to limited and clearly defined questions. The endocrinologically based prediction of the course of a metabolic syndrome as well as the use of biomarkers for the prediction of treatment response for immunoadsorption in dilated cardiomyopathy are to be named here as respective examples. Obviously, both examples can be understood as examples for the ethical problems of marginal utility, but they certainly give no reason for an alarmist self-promotion of bioethics. Even if one concedes that the alarm calls off bioethics have been provoked through excessive promise of healing and output of IM-propagators and stakeholders in medicine and human biology, it has to be noted that the current status of research raises a very differentiated and, in parts decidedly, clear-cut record: The biomarker-based IM is - according to the thesis of the authors - certainly not the new paradigm of medicine, but a research approach, which may contribute progress in very limited areas in medical care.

